# Is scenery mandatory for restoration? Attention restoration without visual nature elements

**DOI:** 10.3389/fpsyg.2025.1556672

**Published:** 2025-05-01

**Authors:** Hiroto Sakimura, Tomoko Sugawara, Kohta Watatsu, Riho Watanabe, Keiko Tanaka, Akira Wakana, Koji Konuma, Yasuhiko Niimi, Tetsuo Kurahashi, Hiroyuki Sakai, Katsunori Kohda, Nobuhiko Muramoto

**Affiliations:** ^1^Toyota Central R&D Labs., Inc., Nagakute, Japan; ^2^Toyota Motor Corporation, Toyota, Japan; ^3^DENSO CORPORATION, Kariya, Japan

**Keywords:** Attention Restoration Theory, indoor environment, multisensory, Perceived Restorativeness Scale, electrodermal activity, heart rate variability, cognitive performance

## Abstract

**Introduction:**

This study examines the contribution of non-visual nature elements in attention restoration, addressing a gap in research that often prioritizes visual stimuli. While previous studies emphasize visual components, this research investigates whether attention restoration can occur in the absence of visual input.

**Methods:**

A within-subject experiment involving 47 participants compared three conditions: a multisensory nature-like environment (visual, auditory, olfactory, and tactile stimuli), a similar environment without visual stimuli, and a control condition with no nature-like stimuli.

**Results:**

A discrepancy between subjective and objective measures was observed. Although self-reported restoration was improved by the existence of visual stimuli, both multisensory nature-like conditions promoted significant physiological benefits (parasympathetic activation and sympathetic deactivation were indicated from heart rate variability and electrodermal activity) with no substantial differences between the presence or absence of visual stimuli. No statistical significance was found in cognitive measures among all conditions.

**Discussion:**

These findings challenge the vision-centric paradigm of restorative environments and highlight the potential of auditory, olfactory, and tactile stimuli to independently foster physiological recovery. By incorporating multisensory elements of nature, this study underscores the importance of non-visual modalities in restorative design. Practical implications include the development of restorative environments for urban spaces or healthcare settings where visual access to nature is limited.

## 1 Introduction

Our attention, a limited cognitive resource, is easily depleted by prolonged or demanding tasks (Cohen and Spacapan, 1978; Muraven et al., 1998; Muraven and Slessareva, 2003; Berman et al., 2008; Bratman et al., 2012). Recognizing how to replenish this resource is essential for our well-being, and natural environments have long been acknowledged for their restorative qualities. Kaplan's Attention Restoration Theory (ART) provides a useful framework by highlighting the cognitive benefits of these natural settings (Kaplan and Kaplan, 1989; Kaplan, 1995). ART distinguishes between two types of attention: directed attention, which requires effort and eventually leads to fatigue, and involuntary attention (fascination), which occurs effortlessly and aids in recovery (James, 1892; Berto, 2005). Directed attention is necessary for focus-intensive tasks, whereas involuntary attention, often evoked by nature, helps restore cognitive resources.

According to ART, four characteristics—Fascination, Being Away, Extent, and Compatibility—make a natural setting restorative. Together, these components replenish attentional resources and enhance cognitive performance. To evaluate environments based on their restorative potential, various versions of the Perceived Restorativeness Scale (PRS) have been developed (Laumann et al., 2001; Berto et al., 2008; Rhee et al., 2023; Shibata et al., 2024). In addition to cognitive benefits, restorative environments also offer physiological advantages, providing alternative indicators of environmental impact (Berto, 2014; Grassini et al., 2019; Chen et al., 2020; Kim et al., 2021).

Although extensive research supports the cognitive benefits of natural environments (Tennessen and Cimprich, 1995; Hartig et al., 1996, 2003; Laumann et al., 2003; Bratman et al., 2012; Atchley et al., 2012), most studies focus primarily on visual stimuli. Visual elements -such as landscapes, greenery, and nature videos- are often considered the main contributors to the attention restoration. PRS assessments of environmental restorativeness also emphasize visual properties, and many studies use photographs to present target environments to participants (Van den Berg et al., 2014; Li et al., 2022; Shibata et al., 2024).

However, this visual-centric approach overlooks other sensory modalities, such as auditory, olfactory, and tactile stimuli, which may also contribute to restoration. Recent research on virtual and simulated natural environments suggests that immersive experiences can yield similar benefits to real nature, hinting that non-visual stimuli might also support attention restoration (Browning et al., 2020; Aristizabal et al., 2021; Ojala et al., 2022; Takayama et al., 2022). Although some studies have examined auditory, olfacory, and tactile contributions to restoration (Alvarsson et al., 2010; Koga and Iwasaki, 2013; Hedblom et al., 2019; Michels and Hamers, 2023), the psychological, physiological and especially cognitive benefits of non-visual elements remain underexplored. These findings imply that visual stimuli alone may not fully account for the restorative effects of nature.

Moreover, previous studies have largely examined unimodal effects, particularly for non-visual aspects, thereby neglecting the inherently multisensory nature of natural environments. The combined effects of non-visual stimuli remain unclear. Although the effect of cross-modal effects has been intensively studied in the context of cognitive psychology (Brosch et al., 2009; Evans, 2020; Rienäcker et al., 2020; Del Popolo Cristaldi et al., 2023), most of these studies are limited to bimodal effects. In particular, the main interest of such studies is how visual perception is affected by stimuli from other modalities, thus the accumulated knowledge is vision-centric. Limiting research to visual elements leaves substantial gaps in our understanding of how different sensory mechanisms collectively contribute to restoration.

The vision-centric paradigm on ART leads a question: Are visual stimuli mandatory for restoration in a multisensory environment? To address this, our study investigates the contribution of non-visual sensory elements in attention restoration. We hypothesize that it is possible to induce attention restoration without visual elements of nature. In other words, visual elements of nature are not indispensable to induce attention restoration.

Using a within-subject experimental design, we examine how different multisensory stimuli (with and without visual components) affect attention restoration. Our findings aim to challenge the prevailing assumption that visual stimuli are fundamental to attention restoration, underscoring the potential of non-visual aspects of nature to facilitate restoration. Ultimately, this research seeks to expand ART by adopting a more comprehensive perspective on the sensory elements that support recovery in natural environments.

## 2 Materials and methods

### 2.1 Overview of study design

**Figure 1:**
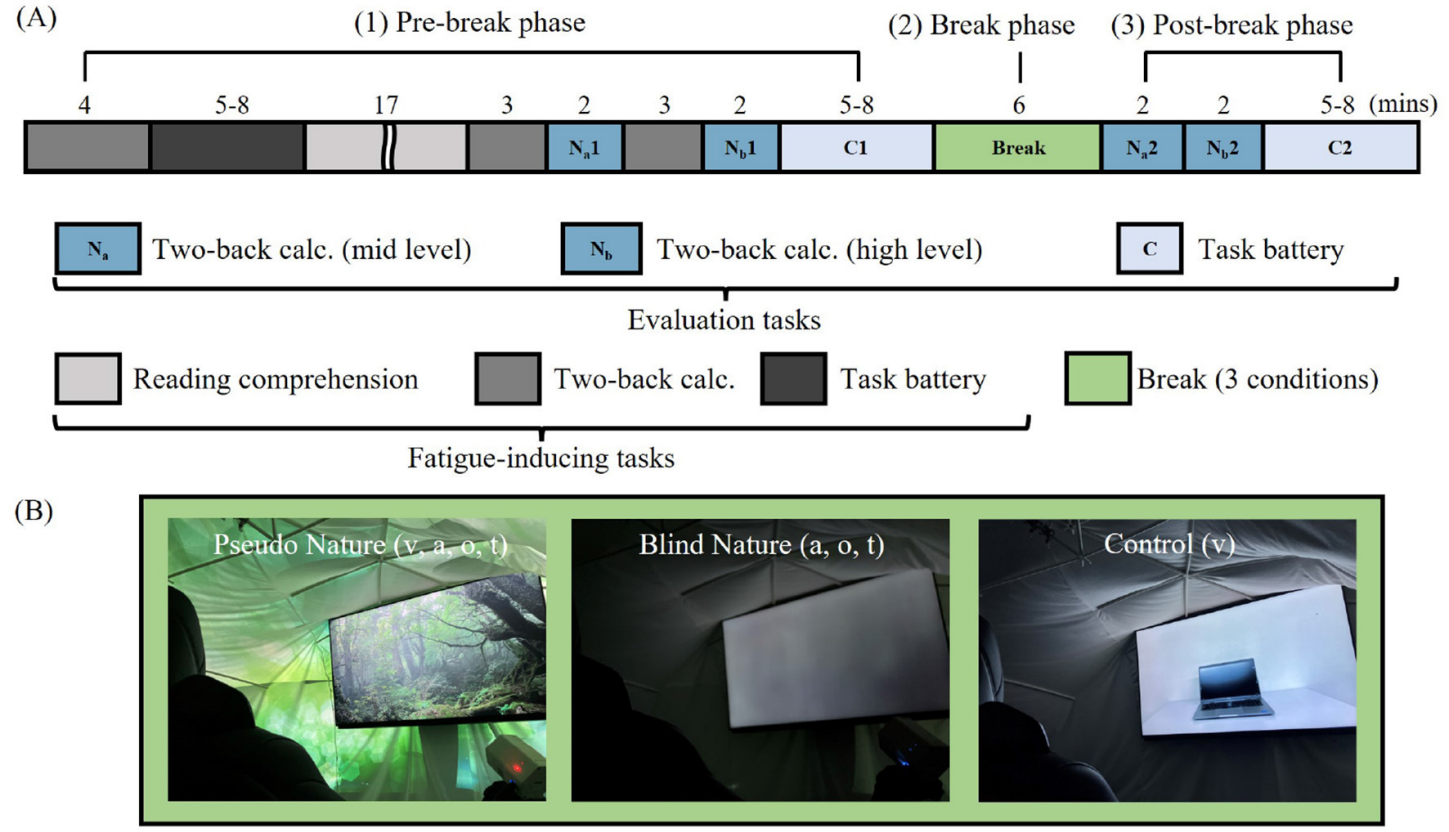
**(A)** Experimental protocol and task details. **(B)** Images during the Break phase for each condition. The letters v (visual), a (audio), o (olfactory), t (tactile) correspond to presented elements of stimuli in each condition. The tactile stimuli combined wind from the fan and warm air from the blowers, stimulating both pressure sensation and warmth perception.

Our key hypothesis is that “visual information of nature is not indispensable to induce attention restoration.” To test this, our research used an within-subject design to investigate the contribution of visual information to attention restoration. The experiments were carried out at Toyota Central R&D Labs., Inc., from November 2022 to February 2023. The participants attended the lab thrice, each time undergoing a different experimental condition. An experiment lasted approximately 60 minutes, divided into three phases as depicted in Figure 1A: (1) Pre-break phase, where the participants performed fatigue-inducing tasks and evaluation tasks for approximately 33 and 10 min per each. They were expected to maintain a directed attention state (visual-input-oriented) by engaging these tasks which were totally given by visual information; (2) Break phase, involving a 6-minute break with environmental stimuli; and (3) Post-break phase, the participants performed evaluation tasks again for about 10 min to quantify their cognitive abilities after the Break phase.

We set three conditions for the Break phase, which were designed to unveil the contribution of non-visual information of nature to attention restoration by following the standard experimental design of ART, which compares nature to artificial urban/office environment. To mitigate order effects, the sequence of experimental conditions was randomized.

The three experimental conditions for the Break phase were the following:

Pseudo Nature Condition (PN) : Designed to simulate a natural environment, the participants experienced a multisensory stimulus package that included a forest video with the corresponding sounds (including the surrounding projection and sound of the hypersonic band ), scents, and wind.
Blind Nature Condition (BN) : Designed to test the impact of non-visual information of nature on attention restoration, the participants were exposed to the same auditory, olfactory, and tactile stimuli as PN in the dark. In other words, the visual stimuli (video and surrounding projection) were excluded from PN.
Control (C) : Designed to work as the Control designed by following typical experimental conditions of ART (Ojala et al., 2022; Rhee et al., 2023), the participants viewed an image of a laptop placed on a desk throughout the Break.

Figure 1B shows the stimuli for each condition. All stimuli lasted for 6 min, covering the entire Break phase whose duration was determined from (Browning et al., 2020). The same difficulties of tasks were given to the participants regardless of the conditions during the Break phase.

Most studies on ART involve natural settings, such as forests and plants, or simulated environments through images and videos (Tennessen and Cimprich, 1995; Hartig et al., 1996, 2003; Laumann et al., 2003; Berto, 2005; Bratman et al., 2012; Atchley et al., 2012; Hedblom et al., 2019; Aristizabal et al., 2021; Ojala et al., 2022; Takayama et al., 2022; Rhee et al., 2023). These investigations largely depend on visual cues to facilitate attention restoration, with noted success. PN was expected to produce similar restorative effects as in these previous studies. Our hypothesis suggests that restoration does not hinge solely on visual stimuli; rather, non-visual stimuli from nature can also play a significant role. BN aims to test this by removing visual stimuli. The same level of restoration potential would be shown for both the PN and BN compared to the Control if our hypothesis was true.

One subjective and two objective (physiological and cognitive) measures were used to assess the restorative property of each condition. Participants' cognitive performance was measured using a variety of cognitive tasks before and after the Break phase, as depicted in Figure 1A (Evaluation tasks). These tasks were specifically designed to keep participants' attention focused on visual stimuli, presented on the monitor. No other modalities were used for task input, ensuring that attention remained fixed on the visual elements during both Pre- and Post-break phases. To guarantee the consistency of each environment, all stimuli in the experiment were presented by programmed electronic devices, and the whole experimental process was controlled electronically.

### 2.2 Participants

Participants : A total of 47 full-time healthy knowledge workers (all of them were working in Aichi prefecture in Japan) volunteered for this study, including 22 women. The participants were Japanese speakers between the ages of 22 and 39 years (*M* = 30.3, *SD* = 4.3). The nationality of the participants was not checked for this experiment.
Exclusion criteria : Individuals with regular hospital attendance due to diseases, impaired hearing, smell, tactile, vision, or hand movements, and regular smokers were excluded. Participants refrained from alcohol consumption 24 h and eating/drinking 30 min before each experiment.
Informed consent : Before the first experiment, participants received information about the study, their rights, and the goals of the experiments. Written informed consent was obtained. The study was approved by the ethics committee of Toyota Central R&D Labs., Inc.
Sample size : The sample size required to achieve statistically significant main effects in subjective measures and significant interactions in physiological measures was estimated with reference to Hedblom et al. (2019), who employed 154 participants in a between-subjects design to compare three environmental conditions. Their study demonstrated that exposure to multisensory stimuli designed to reproduce green-rich environments significantly influenced skin conductance. In contrast to their study, we adopted a within-subject design, which generally requires fewer participants due to reduced variability. Given the limited resources available for this study, we recruited 47 participants, resulting in a total of 141 samples across three conditions.

### 2.3 Experimental setup

**Figure 2:**
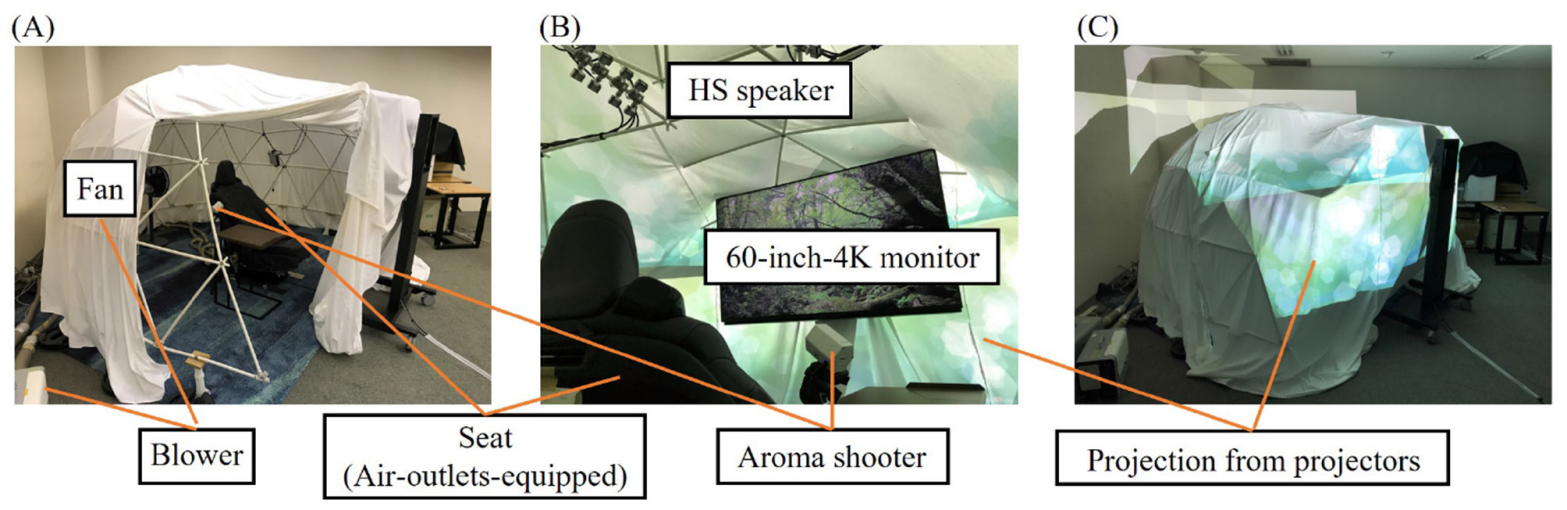
Images of the experimental environment and the placement of each device: **(A)** Outer view without stimuli. Participants remained seated throughout the experiment. **(B)** Inner view of PN (the view from participants). **(C)** Outer view of PN.

We constructed a linen-covered dome equipped with various devices to create a controlled restorative environment, as depicted in Figure 2. We aimed to minimize environmental fluctuations between participants during the experiments. The devices included the following components:

- Visual: A 60-inch 4K monitor for the primary view and projectors for the surrounding view. The video contents was the forest of Yakushima island in Japan as primary view (available on YouTube) and greenery light as surrounding view.
- Auditory: Wireless earphones with noise-canceling functionality. The sound of the video (the sound of forest) was played as the auditory stimulation.
- Olfactory: An aroma shooter from which the flavor of forest was shot. Shooting for 4 seconds and then resting for 6 s were repeated throughout the Break.
- Tactile: A fan and temperature-controllable blowers that emitted warm wind from outlets equipped by the seat.
- Inaudible frequency stimuli: Hypersonic speakers (HS speakers) to produce sound in the frequency range of 20–100 kHz, creating an environment reminiscent of a real forest (Nishina and Oohashi, 2005).

All devices were precisely controlled to present predefined sets of stimuli for each experimental condition. The room temperature was maintained at 23.7 ± 0.9°C throughout all experiments. In this article, we refer to the experimental environments (PN, BN, and Control) based on specific sets of stimuli, using the terms ‘set of stimuli' and ‘environment' interchangeably to describe the conditions during the Break phase of the experiments.

### 2.4 Experimental procedures

In each experiment, participants visited the laboratory and three biosensors were attached: Bitalino, Empatica E4, and Tobii Pro Glasses 3 to measure electrocardiogram, electrodermal activity, and eye movements. The participants then sat in the dome, put on wireless earphones, and began the experimental procedure.

Unlike some previous studies where participants walked in natural settings or moved to separate rooms for the Break phase (Hartig et al., 2003; Ojala et al., 2022), the participants in this study were instructed to remain seated throughout the experiment. They were also advised to avoid body movements that could cause artifacts. This approach aimed to prevent the confounding effects of stimuli on attention restoration from being intertwined with changes in body position (e.g., transitioning from a seated position to standing or walking). This operation minimized the potential mixed effects of body movement and environmental exposure during the break.

In addition, participants were instructed to keep their eyes open during the Break phase, regardless of the condition. This precaution helped to avoid any eye-closing effects (such as parasympathetic nervous system activation). Notably, the specific objectives of each break condition were not disclosed to the participants prior to the experiment.

We scheduled experiments within four time categories: 10:00–12:00 (9:15–11:15 for two participants), 13:30–15:30, 15:45–17:45, and 18:00–20:00. To maintain consistency in alertness and vital signal behavior (especially heart rate variability (HRV)), each participant underwent three separate experiments within the same time category. A minimum of 48 hours separated experiments for each participant.

### 2.5 Measures

We used three types of measures to assess the recovery effects of each environment: the Perceived Restorativeness Scale (PRS) for subjective evaluation, and physiological measures along with cognitive tasks for objective assessment.

#### 2.5.1 Subjective measures

In this study, we used the Perceived Restorativeness Scale (PRS) (Korpela and Hartig, 1996; Hartig et al., 1997a,b) to assess the subjective restorative quality of each environment during the Break phase. The PRS was developed to capture participants' intuitive sense of restoration related to their surrounding environmental conditions (Hartig et al., 1997a; Purcell et al., 2001). We used an 11-point Likert scale (ranging from 0 = “not at all” to 10 = “completely”) to evaluate each factor. After completing their last experiment, participants were individually exposed to each break condition once again to score the PRS for all three environments.

Additionally, we used the NASA Task Load Index (NASA-TLX) (Hart and Staveland, 1988), which is the subjective measure of workload, to evaluate fatigue levels during the Fatigue-inducing tasks across the three experimental conditions. Following each experiment, participants provided subjective ratings of their fatigue levels during fatigue-inducing tasks using the NASA-TLX assessment.

#### 2.5.2 Physiological measures

In this study, we analyzed HRV, skin conductance, and eye movements to capture autonomic nervous activity (Shaffer and Ginsberg, 2017; Bafna et al., 2021). Prior to the analysis, we visually inspected the cleaned data. The dataset was segmented into non-overlapping 2-minute segments to focus on representative values within each 2-minute interval.

Specifically, for HRV, we examined both time domain and frequency domain measures. The former included standard deviation of normal-to-normal RR intervals (SDNN), root-mean square of successive RR interval differences (RMSSD), and the Cardiac Deceleration Index (CDI) calculated from the Poincaré plot (Toichi et al., 1997; Allen et al., 2007). The latter consisted of power at low frequencies (LF, 0.04–0.15 Hz) and high frequencies (HF, 0.15–0.4 Hz). In total, five HRV metrics were analyzed. Furthermore, we extracted the variation of electrodermal activity (EDA) from E4 and pupil diameter (PD) from eye-tracking data recorded using Tobii Pro Glasses 3 to evaluate autonomic nervous activities.

#### 2.5.3 Cognitive measures

We used several types of tasks, which were categorized into two roles: evaluation tasks to assess cognitive functions and fatigue-inducing tasks to induce fatigue. Prior to their first experiments, all participants received instructions for all tasks and practiced until they understood the rules and how to answer. Consequently, we do not anticipate any training effects. For the evaluation tasks, we used two types of tasks to measure cognitive performance (depicted in blue in Figure 1A):

- Two-Back Calculations: We used two different levels of two-back calculations (*N*_*a*_ and *N*_*b*_ in Figure 1A) to evaluate working memory.
- Task Battery: Participants completed a battery of cognitive tasks provided by CogEvo (Total Brain Care, Kobe, Japan), which has been shown to have the potential to distinguish early-stage cognitive impairment (Takechi and Yoshino, 2021). From this battery, we selected five tasks to assess four cognitive functions: memory, attention, spatial perception, and executive function.

The detailed descriptions of each task are provided in the Appendix, where details of the fatigue-inducing tasks are also explained.

### 2.6 Data analysis

We performed data analysis using R software (version 4.2.2) (R Core Team, 2018). Descriptive statistics were computed for each variable and normality assumptions were assessed using the Shapiro-Wilk test. To compare task performance and physiological metrics across break conditions and phases, we employed repeated measures (RM) two-way ANOVA.

Specifically, the task performance was compared between Evaluation task scores of the Pre-break and Post-break phases. And physiological metrics were compared between values at the end of Pre-break and whole Break phases (averaged over the 6 minutes before the Break, denoted as “C1” in Figure 1A, and the 6-minute average during the Break phase). The RM two-way ANOVA was performed using ANOVA-kun (version 4.8.7) (Iseki, 2023). We set the significance level at .05 for all statistical tests.

Some participants were excluded from the ANOVA due to incomplete data, which was induced by unavoidable accidents, such as sensor misalignment by body movement during experiments. The available samples are denoted in each ANOVA result in the next section. We also performed analysis with linear mixed-effect models to eliminate the effects of these missing data, whose details and results are described in the Supplementary material.

## 3 Results

### 3.1 Subjective measures

None of the six factors in the NASA-TLX, which were used to evaluate stress levels during fatigue-inducing tasks, showed a significant difference among break conditions (*p*>0.05). This result implies that the same level of fatigue was successfully induced by the fatigue-inducing tasks regardless of the break conditions, thereby validating the subsequent discussions.

The PRS results are presented in Table 1. Holm's sequentially rejective Bonferroni *post hoc* tests after repeated-measures one-way ANOVA revealed that the restorative quality of PN and BN was significantly higher for “Being Away”, “Fascination”, “Compatibility”, “Preference”, and “Familiarity” compared to the Control. Additionally, the PRS scores of PN were significantly higher than those of BN across all the aforementioned factors. In contrast, the subjective restorative quality of PN and BN was significantly lower in “Coherence” than that of the Control. In total, the restoration property of each condition was ranked as PN>BN>C (the sum of all factors in PRS, *p* < 0.05) according to PRS.

**Table 1:** Mean score, standard deviation (in the parentheses) and the repeated-measures ANOVA results [F_(2,92)_ and *p*-value] of PRS for each break condition (*N* = 47).

	**PN**	**BN**	**Control**	***F***	***p***
Being away	6.95 (1.97)^*a*^	5.74 (2.17)^*b*^	2.40 (1.72)^*c*^	84.72	< 0.001
Fascination	6.15 (1.98)^*a*^	3.79 (1.72)^*b*^	1.26 (1.33)^*c*^	116.2	< 0.001
Scope	5.39 (1.73)^*b*^	2.73 (1.62)^*c*^	2.64 (1.71)^*c*^	56.50	< 0.001
Coherence	3.63 (1.77)^*b*^	1.96 (1.70)^*c*^	4.39 (2.00)^*a*^	41.68	< 0.001
Compatibility	4.76 (1.45)^*a*^	3.93 (1.67)^*b*^	3.11 (1.39)^*c*^	20.52	< 0.001
Preference	5.54 (2.00)^*a*^	3.81 (2.31)^*b*^	1.42 (1.54)^*c*^	67.59	< 0.001
Familiarity	3.89 (2.71)^*a*^	2.10 (1.87)^*b*^	0.787 (1.21)^*c*^	37.87	< 0.001

Results of *post hoc* tests performed with Holm's sequentially rejective Bonferroni correction: *a*>*b*>*c*.

### 3.2 Physiological measures

**Figure 3:**
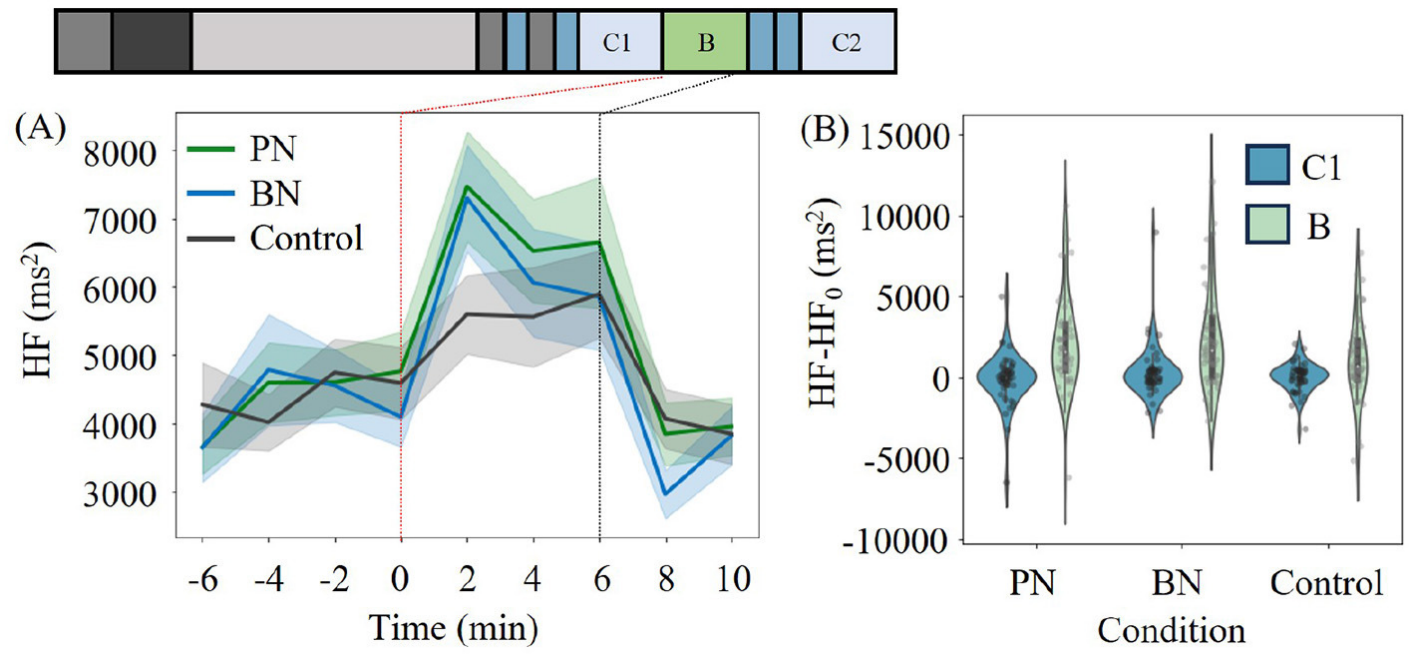
**(A)** The time series of average HF of all participants for each condition. The filled-color region stands for the standard error. **(B)** The 6-min average distribution during C1 and Break **(B)** for each condition. The value at the end of C1 or the beginning of the Break phase, which is plotted at 0 in **(A)**, was set as zero for each experiment. White points in the violinplots indicate the median and the gray dots represent the individual data points.

Figure 3A displays the time series of mean HF averaged for all participants around the Break phase, categorized by each break condition. HF was calculated with a two-minute window without overlapping, as explained in Method. The standard error (SE) is represented by the filled-color region in the plot. The start point of the Break phase (or the end of C1) is designated as zero minutes (indicated by the red line) for convenience. In particular, the mean change in HF exhibits distinct transition patterns during the Break phase, depending on the stimuli conditions. Furthermore, PN and BN induced a greater variation in HF, particularly during the initial two minutes of the Break phase.

As the data preprocessing for the statistical analysis, we subtracted the HF value at time 0 (the HF value at the end of C1, denoted HF_0_) from all other observed values within the same experiment to focus on the mean transition of HF for each participant. In other words, we set the HF value at time 0 as the baseline (HF_0_) for each experiment. The 6-minute average HF during C1 and the Break (averaged over three data points in each phase) were compared, as shown in Figure 3B. The gray dots in Figure 3B represent the individual data points.

We conducted RM two-way ANOVA to elucidate the effect of each stimulus set on HF. Several significant differences emerged. First, there was a main effect of phase (6-minute average during C1 and the Break), indicating an increase in HF during the Break across all conditions. Furthermore, the interaction effect of phase and conditions was also significant. *Post hoc* analysis revealed that the increase in HF was more pronounced for PN and BN compared to the Control. There was no significance in low-frequency (LF) HRV.

**Figure 4:**
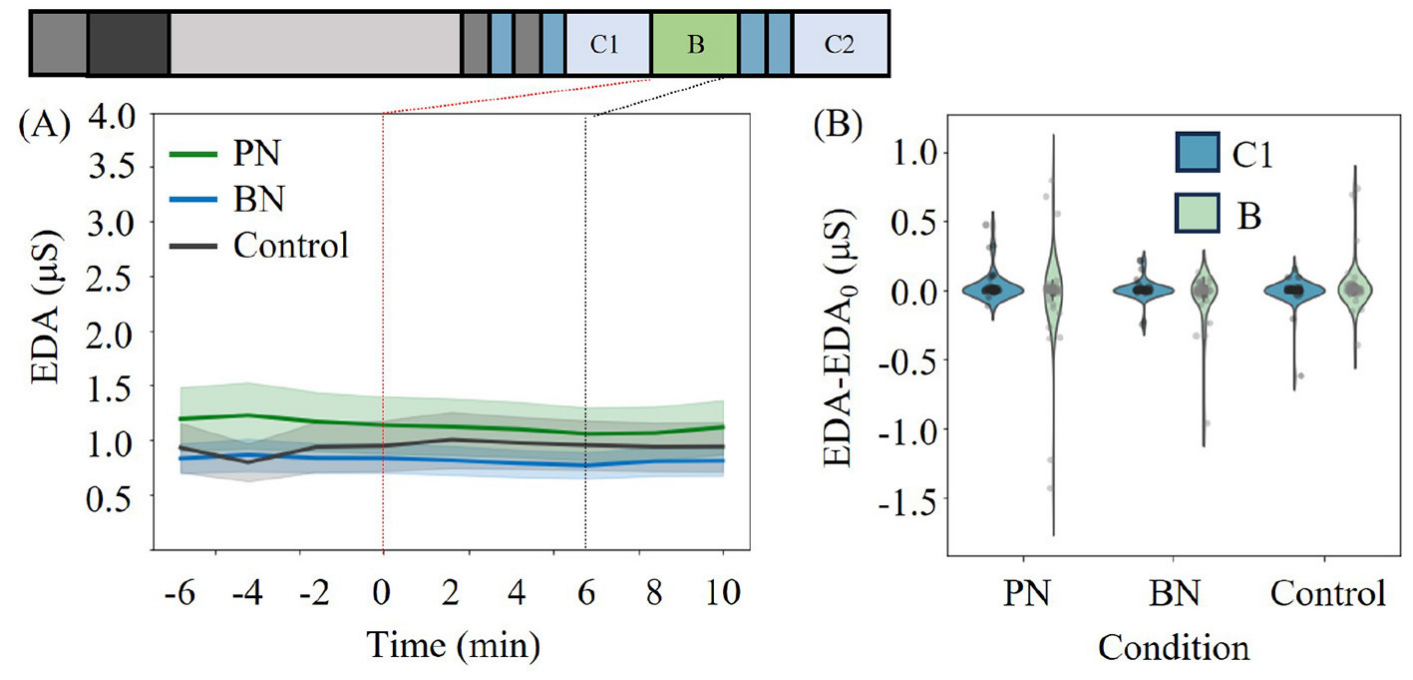
**(A)** The time series of average EDA of all participants for each condition. The filled-color region stands for the standard error. **(B)** The 6-min average distribution of EDA during C1 and Break **(B)** for each condition. The value at the end of C1 or the beginning of Break phase, which is plotted at 0 in **(A)**, was set as zero for each experiment. White points in the violinplots indicate the median and the gray dots represent the individual data points.

Furthermore, in the electrodermal activity (EDA), which is believed to reflect sympathetic nervous system activation, there was a significant decrease in PN and BN compared to the Control (N=35 due to missing data, *F*(2, 68) = 7.343, *p* = 0.001, partial η^2^ = 0.178 for RM two-way ANOVA), as shown in Figures 4A, B. This result implies the sympathetic nervous system activity was more suppressed in PN and BN than that of Control.

Table 2 summarizes all the indices tested in this study. Participants who included missing data points were excluded from the analysis. We performed RM two-way ANOVA in the same manner as performed above. Overall, we found significant interaction effects between conditions and phases in HF and CDI, which reflect the activeness of parasympathetic nerves. While no significance was found in SDNN and RMSSD, the participants' physiological states were more inclined toward a parasympathetic-nervous-active state under PN and BN. EDA showed a significant decrease in PN and BN compared to the Control, which can be interpreted as the suppression of sympathetic nervous system activity for PN and BN. In total, the restoration property of each condition can be ranked as PN>C and BN>C. Notably, no significant difference was observed between PN and BN according to physiological measures, which is inconsistent with the result of PRS (PN>BN>C). The results of LMM did not show any discrepancy with ANOVA within these measures. The details of LMM analysis are summarized in the Supplementary material.

**Table 2:** Results of interaction effects from RM two-way ANOVA for HRV metrics (*N* = 47) and *post hoc* tests performed with Holm's sequentially rejective Bonferroni correction: ^*^p < 0.05.

	**F_(2,92)_**	***p***	**Partial η^2^**	***Post hoc***
SDNN	2.176	0.119	0.045	-
RMSSD	2.707	0.072	0.056	-
CDI	3.195	0.045^*^	0.065	PN>C, BN >C
HF	4.549	0.013^*^	0.090	PN>C, BN >C
LF	1.170	0.315	0.025	-
EDA	7.343^*a*^	0.001^*^	0.178	PN < C, BN < C

^*a*^ represents F_(2,68)_, *N* = 35 due to missing data.

It should be noted that the posture of the participants remained static between the Pre-break and Break phases. Thus the variation of physiological matrices described above can be fully attributed to the change of environment. The results of pupil diameter measurements, which is thought to reflect the activity of the autonomic nervous system, were eliminated because of the difficulty to separate the pure contribution of autonomic nervous activity due to the difference in illuminance among the break conditions (PN = 32.6, BN = 0.4, and Control = 19.5 lx.

### 3.3 Cognitive measures

We employed two types of evaluation tasks: a task battery (CogEvo) and two-back calculations. Table 3 summarizes the scores for five tasks in the task battery, along with the results of the RM two-way ANOVA (including the F and *p*-value of the interaction effect) for each break condition (*N* = 47). We found no interaction effect in the scores between the Pre- and Post-break phases, nor between the different break conditions (PN, BN, and Control). Furthermore, there was no significant main effect observed for either phase or break condition. These findings suggest that no change of cognitive performance occurred before and after the Break phase, or no restoration was observed (all *p*-values tested in this scheme were above 0.1). The lack of recovery might be attributed to the task battery's relative ease, making it difficult for participants in this experiment to detect fatigue and restoration (Ohly et al., 2016), or the task performance was not influenced by the fatigue accumulated in the Fatigue-inducing phase.

**Table 3:** Scores of five cognitive tasks before and after the Break phase and the results of RM two-way ANOVA [ F_(2,92)_ and *p*-value for interaction] for each break condition (*N* = 47).

	**PN**		**BN**		**Control**		**Interaction**	
	**Before**	**After**	**Before**	**After**	**Before**	**After**	*F*	*p*
Attention 1	291 (78.3)	308 (72.2)	316 (71.6)	315 (73.6)	309 (75.2)	299 (78.9)	0.733	0.483
Attention 2	289 (40.2)	297 (38.3)	294 (34.4)	294 (35.7)	296 (31.3)	302 (33.8)	0.459	0.633
Memory	552 (187)	601 (191)	567 (207)	604 (173)	531 (207)	550 (228)	0.234	0.791
Executive function	307 (58.4)	316 (53.9)	292 (56.3)	291 (68.6)	301 (65.6)	316 (56.7)	0.613	0.544
Spatial perception	445 (70.7)	445 (75.6)	443 (63.4)	441 (72.3)	443 (81.9)	420 (111)	0.705	0.497

**Figure 5:**
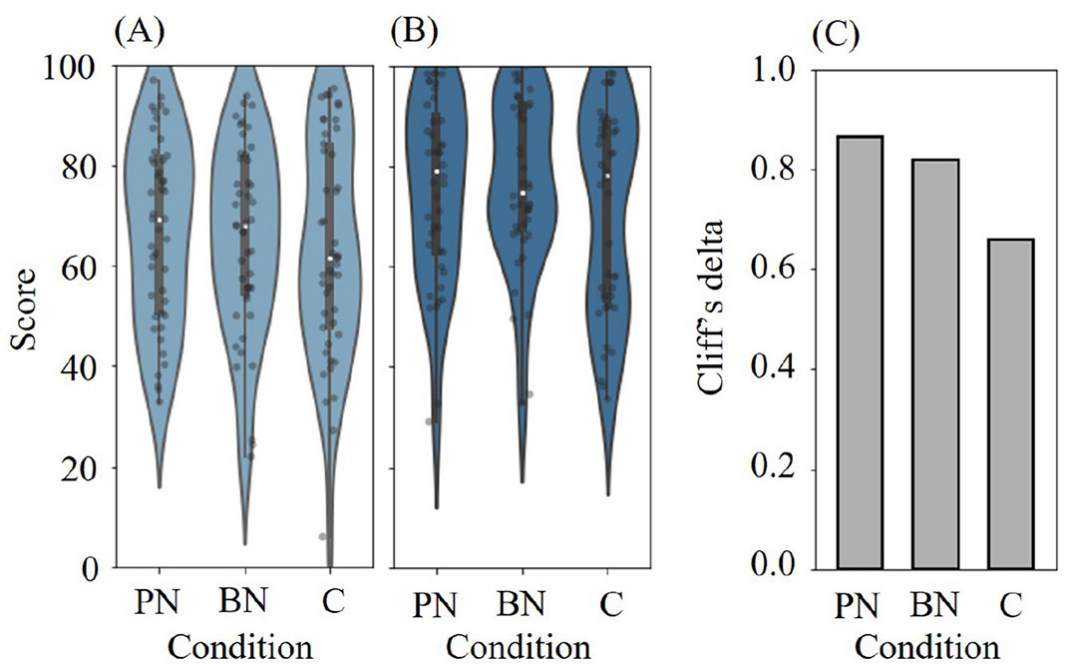
Score distributions of two-back calculations at **(A)** Pre- and **(B)** Post-break phase for each condition (*N* = 44). White points in the violinplots indicate the median. **(C)** Cliff's delta between scores at Pre- and Post-break phases for each condition. C refers to Control.

Figures 5A, B depict the average scores for the two difficulty levels of two-back calculations at the Pre- and Post-break phases for each condition (*N* = 44). Unfortunately, three data points were missing due to a system malfunction, so we removed the data from those participants during the analysis.

We conducted a RM two-way ANOVA between the stimuli conditions and phases. The results showed a significant difference in the main effect of the phase factor (F_(1,43)_ = 44.13, *p* < 0.001, partial η^2^= 0.506). However, no significant difference was found in the main effect of conditions (*F*(2, 86) = 1.994, *p* = 0.142, partial η^2^=0.044) or the interaction effect between phase and condition (*F*(2, 86) = 0.606, *p* = 0.548, partial η^2^=0.014). This result indicates that there was a significant difference in score variation regardless of the break conditions.

Although no significant interaction effect was detected, we conducted multiple comparisons using non-parametric methods (specifically, the Wilcoxon signed-rank test with Holm-Bonferroni correction). This choice was made because the score distributions for Post-break phase was confirmed to be non-parametric based on the Shapiro-Wilk test (*p*=0.022, 0.011, and 0.001 for PN, BN and Control). The results showed no significance (*p* = 0.090 between PN and Control, *p* = 0.086 between BN and Control after correction by Holm-Bonferroni method. *p* = 0.910 between PN and BN).

For further evaluation, we calculated the effect size of the score transition between the Pre- and Post-break phases. We employed Cliff's delta since the score distribution was non-parametric. The calculated Cliff's delta values were 0.864, 0.818, and 0.659 for PN, BN, and Control, as plotted in Figure 5C. In addition, 38, 36, and 29 out of the 44 participants showed score increase in two-back calculations after the Break phase for PN, BN, and Control, respectively. No significant difference was observed among cognitive performance. However, the effect size of score transition was larger for PN and BN than that of Control. This relation is closer to physiological indicators' (PN>C and BN>C) than that of PRS (PN>BN>C).

## 4 Discussion

The present study aimed to test whether the visual information of nature is mandatory for the subjective and objective (physiological and cognitive) restorative effects after the induction of cognitive stress. To test this hypothesis, we compared three conditions: Pseudo nature (PN, full set of environmental stimuli), Blind nature (BN, PN without visual stimuli), and Control.

Results showed a notable inconsistency between subjective and objective measures of restoration. According to subjective ratings using the PRS, the absence of visual stimuli diminished the perceived restorative quality of the environment. In contrast, objective (physiological and cognitive) measures indicated that removing visual properties did not significantly impact restorativeness. Especially, both PN and BN produced nearly equivalent effects in the physiological indices tested in this study, although no significance was found in cognitive indicators. The vision-centric focus of the PRS (Shibata et al., 2024) may explain this discrepancy, highlighting the need to consider non-visual contributions to restoration beyond subjective visual assessments.

These findings suggest that visual stimuli are not strictly necessary for physiological restoration in multisensory environment, even though the participants subjectively perceived environments without visual input as less restorative. This discrepancy between subjective and physiological outcomes highlights the complex nature of attention restoration mechanisms. The contribution of non-visual stimuli (especially the olfactory) to stress reduction is also emphasized in Hedblom et al. (2019), which provides same indication with our current result. Together with the presented study, a concrete evidence indicating the significance of non-visual properties in nature on attention restoration is shown. We discuss the potential mechanisms of attention restoration and limitations in the following.

### 4.1 Mechanisms of attention restoration

One potential mechanism underlying attention restoration in the absence of visual stimuli is the capacity of other sensory cues -such as auditory, olfactory, and tactile stimuli- to evoke natural experiences. Recent studies imply the top-down nature of attention restoration as tested in Koivisto et al. (2022); Koivisto and Grassini (2024). In this framework, it can be inferred that non-visual senses may have compensated for the lack of visual input, promoting recovery through the mental imagery and emotional associations they elicited in BN. The fact that PN and BN resulted in the same level of physiological and cognitive restoration can be interpreted as that they were the trigger for the participants to invoke same level of imagery state of nature, resulted in the same level of restoration effect. Our results may support the top-down mechanism of attention restoration by nature.

Additionally, we propose a “modality releasing (MR)” mechanism, in which directing attention away from overused sensory channels (here, vision) toward other modalities can facilitate recovery through the activation of corresponding brain regions by shifting attention (Spence, 2002; Salmi et al., 2009; Keller, 2011). This leads to the relative deactivation of the brain regions to process the overused sensory channels, resulting in the restoration. In this study, tasks were designed to engage the participants' visual attention exclusively, ensuring that during both Pre- and Post-break phases, attention remained fixed on visual elements. Therefore, the restoration observed here may stem from this modality-releasing effect. MR may provide a new interpretation of the “soft fascination,” whose definition remains ambiguous (Joye and Dewitte, 2018).

Although MR might appear inconsistent with prior studies that achieved attention restoration through purely visual means (e.g., viewing pictures) (Berto, 2005; Berman et al., 2008; Gamble et al., 2014), both methods may operate similarly by diminishing directed attention to visual input and enabling a state akin to involuntary attention. In essence, decreasing attention allocation to the overused sensory channel is the key to induce attention restoration. MR acts as an intermodal method, reducing attention allocation to the overused channel by presenting stimuli to other sensory channels. And visual stimuli from nature can work as intramodal method which reduces attention allocation to visual input by allowing the subject to have nothing to focus on. The idea of MR might open new possibilities for designing restorative environments that balance multiple sensory inputs.

### 4.2 Limitations of this study

Despite its contributions, the present study has several limitations. First, the discrepancy in restoration characteristics between subjective and physiological measures observed in this study may be specific to the measures used. The physiological indicators chosen in the study are not sufficiently accurate and valid to discriminate between the two conditions. We must be careful that the lack of discrimination in HRV and EDA does not allow to be concluded that there are not physiological evidences of a difference between the conditions with and without visual inputs. Furthermore, individual sensory preferences can also influence the result. While some participants may have responded well to auditory, olfactory, or tactile stimuli, others may have required visual input for optimal recovery. This point might be influenced by cultural factors which were not accounted for in this study. Future research should examine these factors to better understand individual differences in sensory-driven recovery.

Second, this study focused on forest-based stimuli, following common representations in digital nature research (Markwell and Gladwin, 2020; Takayama et al., 2022; Reese et al., 2022). While forests are widely used to examine attention restoration, natural environments vary widely (e.g., oceans, lakes, deserts), and each might uniquely impact restoration. Future research should explore diverse natural settings to understand how different environments contribute to attention restoration.

Third, the effect of darkness presented in the BN should be clarified more. The visual stimuli were completely removed in BN. While darkness is often associated with sensory deprivation, it may enhance recovery by reducing cognitive load and encouraging attention to shift toward other sensory modalities. This finding suggests that dark environments, combined with carefully selected non-visual stimuli, could serve as effective restorative spaces. The darkness might also help participants to evoke natural environment to trigger the top-down process of attention restoration. However, the effect of darkness on attention restoration is not explored, which needs further study in the future.

Despite these limitations, the present study offers valuable insights into the contribution of non-visual stimuli in restoration. It challenges the visual-centric approach in ART by demonstrating that non-visual multisensory inputs alone can facilitate restoration. We suggested two potential mechanisms to explain the presented results, top-down process as well as modality releasing. This finding expands the scope of ART and suggests that restorative environments can be designed with a multisensory perspective.

Finally, the study provides practical insights for designing environments where visual access to nature is limited, such as offices without windows, underground spaces, or medical facilities. By incorporating non-visual elements -such as soundscapes, scent diffusers, or tactile features- such spaces can still promote recovery and well-being.

## 5 Conclusion

In conclusion, this study advances our understanding of attention restoration by demonstrating the restorative potential of non-visual sensory inputs. The lack of visual information of nature in a multisensory environment degraded subjective restorative properties, but did not degrade physiological restorative indicators based on HRV and EDA. These findings broaden the scope of ART and underscore the importance of designing multisensory environments to promote attention restoration. And practical implications include the development of restorative environments for urban spaces or healthcare settings where visual access to nature is limited. Future research should investigate cultural and individual characteristics, natural situations other than forest, and the effect of darkness on restoration to further refine our understanding of how natural and simulated environments support attention restoration.

## Data Availability

The datasets presented in this article are not readily available because the data that support the findings of this study are proprietary to Toyota Central R&D Labs., Inc., Toyota Motor Corporation, and DENSO CORPORATION, and therefore, are not publicly available. However, further information about the data may be provided by the authors upon reasonable request, subject to the data-sharing policies and confidentiality requirements of the organizations. Requests to access the datasets should be directed to Hiroto Sakimura, e1774@mosk.tytlabs.co.jp.

## Appendix: Description of cognitive tasks

The details of the cognitive tasks imposed on the participants are described.

### Evaluation tasks

CogEvo (task battery) and two-back calculation was used to evaluate cognitive functions during the experiment.

CogEvo has been shown to have the potential to distinguish early-stage cognitive impairment (Takechi and Yoshino, 2021). Although it has a possibility to score several types of cognitive functions, it has not been clarified whether it has enough resolution to compare the cognitive abilities within healthy people. Since the tasks included in this task battery might be too easy for the participants in this study, we used the two-back calculation as a higher difficulty task to evaluate the cognitive functions of healthy adults.

The two-back calculation test is a combination of the well-known two-back task and one-digit number calculations. The addition and multiplication problems of one-digit numbers were displayed one by one (e.g. 2+9, 3 × 6). The problems were presented one at a time for 3 seconds each, and automatically moved on to the next problem. The participants answered by selecting the answer to the problem shown two questions earlier from twelve choices. It required participants to perform mental arithmetic while keeping track of the previous two results. The difficulty of *N*_*b*_ was set higher than that of *N*_*a*_ by changing the display format of the problems.

### Fatigue-inducing tasks

The fatigue-inducing tasks involved reading comprehension of Japanese sentences displayed on the monitor. The participants selected answers from multiple options by clicking. The above-mentioned tasks were also performed to induce fatigue. Especially, two-back calculations were used as the fatigue inducing task just before the evaluation tasks to ensure a smooth transition from fatigue phase to pre-break phase. All tasks were designed to exhibit the same levels of difficulty among three experiments, which was checked by NASA-TLX.
