# Supplementary material for: Is scenery mandatory for restoration? Attention restoration without visual nature elements
Source: Front Psychol. 2025 May 1;16:1556672. doi: 10.3389/fpsyg.2025.1556672 (PMC12082041; doi:10.3389/fpsyg.2025.1556672)
Supplement: Supplementary file 1 [file Data_Sheet_1.pdf]

Supplementary Material

1 ANALYSIS WITH LINEAR MIXED-EFFECTS MODELS

1.1 Objective

In this Supplementary Material, we describe the details of the analysis using Linear Mixed-effects Models (LMM) to examine the physiological data in the study. This analysis was conducted to account for missing data in several participants, which had been excluded in the repeated-measures (RM) two-way ANOVA in the main text.

1.2 Methods

R software (version 4.3.3) (R Core Team (2018)) with ”lme4” (version 1.1-36) for modeling (Bates et al. (2015)) and ”emmeans” (version 1.10.7) for post hoc analysis (Lenth (2025)) packages was used for the data analysis. Modeling by LMM was performed using restricted maximum likelihood estimation (REML) to examine the effects of condition (Pseudo Nature (PN), Blind Nature (BN), and Control), phase (C1(the end of the Pre-break phase) and Break, as shown in Fig.1(A) in the main text), and their interaction on the dependent variables. Participant ID was included as a random intercept to account for individual variability.

To analyze the effects of break conditions and phases on physiological measures, we specified the following LMM:

$$\text{value}_{i,j} = \beta_0 + \beta_1 \text{Cond}_{i,j} + \beta_2 \text{Phase}_{i,j} + \beta_3 (\text{Cond} \times \text{Phase})_{i,j} + u_i + \epsilon_{i,j} \tag{S1}$$

where $\text{value}_{i,j}$ is the dependent variable for participant i at measurement j, which corresponds to each condition. β_0 is the intercept. β_1, β_2, and β_3 represent the fixed effects of condition, phase, and their interaction. u_i is the random intercept for participant i. $\epsilon_{i,j}$ is the residual error term.

Data preprocessing followed the same steps as in the RM two-way ANOVA reported in the main text, focusing on the mean transition of each measure per participant. The model was fitted using 6-minute data from both phases.

1.3 Results and Conclusions

The results for fixed effects (Estimated coefficients (Estimate) with standard error (SE), and $t-$value with degree of freedom (df)) and corresponding post hoc analysis for HF are shown in Table S1. The meaning of each row is as follows.

- Intercept: Baseline value when Condition = Control and Phase = C1.
- Condition PN: Difference in value between PN and Control in C1.
- Condition BN: Difference in value between BN and Control in C1.
- Phase: Difference in value between Break and C1 when Condition = Control.
- PN$\times$Phase: Whether the effect of PN changes between C1 and Break.
- PN$\times$Phase: Whether the effect of BN changes between C1 and Break.

Table S1. Results of fixed effects from LMM (Estimated coefficients (Estimate) with standard error (SE), and $t-$value with degree of freedom (df)) and post hoc analysis for the Break phase performed with Holm's sequentially rejective Bonferroni correction for HF: $^{*}p < .05$, $^{**}p < .01$, $^{***}p < .001$.

	Estimate (SE)	$t(df)$	Post hoc (Break)
Intercept	-2092.2 (858.2)*	-2.438 (805.6)	-
Condition PN	-2498.5 (1195.3)*	-2.090 (765.6)	-
Condition BN	-1550.6 (1194.8)	-1.298 (766.0)	-
Phase	528.8 (166.1)**	3.183 (766.5)	-
Condition (PN)×Phase	592.2 (234.2)*	2.529 (765.6)	PN>C**
Condition (BN)×Phase	466.5 (234.3)*	1.991 (766.3)	BN>C***

The interactions Condition(PN)×phase and Condition(BN)×phase were significant, suggesting that the effect of phase varied depending on the break condition. Post hoc pairwise comparisons were performed using Holm's sequentially rejective Bonferroni correction to examine differences between break conditions. Both PN and BN showed significant differences from Control in the Break phase as noted in TableS1 ($t(df) = -3.19(767), p = .003$ between PN and Control, $t(df) = -3.77(767), p = .0005$ between BN and Control). No significant difference was observed between PN and BN in the Break phase. No significant differences were observed in the post hoc analysis in C1 phase.

The results of fixed effects show that Condition PN had a significantly lower intercept than Control ($p =$ 0.037), which indicates the HF values of PN were significantly lower than those of Control. However, the post hoc results showed no significant difference between conditions in C1 due to the stricter threshold imposed by multiple comparison correction. Thus, it can be concluded that there is no significant difference between the conditions in the C1 phase.

The results for EDA is described in Table S2, a significant decrease was observed in PN and BN compared to the Control ($t(df) = 2.973(726), p = .0091$ between PN and Control, $t(df) = 2.870(720), p = .0091$ between BN and Control). Similarly as in the HF, no significant differences were observed between PN and BN in the Break phase and between conditions in the C1 phase in the post hoc analysis.

Table S3 summarizes the results of interaction effects in the LMM fixed effects and the corresponding post hoc analyses comparing values in the Break phase. We performed LMM in the same manner for all measures. Overall, we found significant differences for SDNN, HF, CDI, and EDA in the Break phase, which reflect parasympathetic and sympathetic nervous activities. SDNN did not show significance in ANOVA performed in the main text, but a significant difference was detected in the post hoc test after LMM, indicating that the inclusion of a random intercept in the model may have contributed to this result.

This does not alter the general interpretation of the results, which indicate that the physiological states of the participants were more inclined toward a parasympathetic-dominant and sympathetic-suppressed state under both PN and BN conditions. In other words, within the physiological indices used in this study, the presence or absence of visual stimuli did not affect physiological recovery. Thus, the results of the LMM analysis were consistent with those of the RM two-way ANOVA.

Overall, the restorative effect of each condition can be classified as PN $>$ C and BN $>$ C, as concluded in the RM two-way ANOVA presented in the main text.

REFERENCES

Bates, D., Mächler, M., Bolker, B., and Walker, S. (2015). Fitting linear mixed-effects models using lme4. *Journal of Statistical Software* 67, 1–48. doi:10.18637/jss.v067.i01

Table S2. Results of fixed effects from LMM (Estimated coefficients (Estimate) with standard error (SE), and $t-$value with degree of freedom (df)) and post hoc analysis for the Break phase performed with Holm's sequentially rejective Bonferroni correction for EDA: $^{*}p < .05$, $^{**}p < .01$, $^{***}p < .001$.

	Estimate (SE)	$t(df)$	Post hoc (Break)
Intercept	-.10194 (.06569)	-1.552 (752.5)	-
Condition PN	.28554 (.09236)**	3.092 (713.5)	-
Condition BN	.20533 (.09192)*	2.234 (713.4)	-
Phase	.02219 (.01263)	1.756 (712.3)	-
Condition (PN)×Phase	-.06025 (.01805)***	-3.339 (712.4)	PN<C**
Condition (BN)×Phase	-.04633 (.01796)*	-2.580 (712.3)	BN<C**

Table S3. Results for interactions of fixed effects from LMM (N=47) and post hoc analysis for the Break phase performed with Holm's sequentially rejective Bonferroni correction: $^{*}p < .05$, $^{**}p < .01$, $^{***}p < .001$.

	Condition (PN)×Phase		Condition (BN)×Phase	
	Estimate (SE)	Post hoc (Break)	Estimate (SE)	Post hoc (Break)
SDNN	1.2243 (1.2485)	PN>C*	2.5496 (1.2497)*	BN>C**
RMSSD	1.4673 (0.7404)*	-	0.3797 (0.7411)	-
CDI	.02376 (.01530)	PN>C*	.03584 (.01532)*	BN>C**
HF	592.2 (234.2)*	PN>C**	466.5 (234.3)*	BN>C***
LF	252.3 (667.1)	-	1038.7 (667.4)	-
EDA	-.06025 (.01805)***	PN<C**	-.04633 (.01796)*	BN<C**

Lenth, R. V. (2025). *emmeans: Estimated Marginal Means, aka Least-Squares Means*. R package version 1.10.7-100003, https://rvlenth.github.io/emmeans/

R Core Team (2018). *R: A Language and Environment for Statistical Computing*. R Foundation for Statistical Computing, Vienna, Austria
